# Long Term Effect of Curcumin in Regulation of Glycolytic Pathway and Angiogenesis via Modulation of Stress Activated Genes in Prevention of Cancer

**DOI:** 10.1371/journal.pone.0099583

**Published:** 2014-06-16

**Authors:** Laxmidhar Das, Manjula Vinayak

**Affiliations:** Biochemistry and Molecular Biology Laboratory, Department of Zoology (Centre of Advanced Study), Banaras Hindu University, Varanasi, India; Yong Loo Lin School of Medicine, National University of Singapore, Singapore

## Abstract

Oxidative stress, an important factor in modulation of glycolytic pathway and induction of stress activated genes, is further augmented due to reduced antioxidant defense system, which promotes cancer progression via inducing angiogenesis. Curcumin, a naturally occurring chemopreventive phytochemical, is reported to inhibit carcinogenesis in various experimental animal models. However, the underlying mechanism involved in anticarcinogenic action of curcumin due to its long term effect is still to be reported because of its rapid metabolism, although metabolites are accumulated in tissues and remain for a longer time. Therefore, the long term effect of curcumin needs thorough investigation. The present study aimed to analyze the anticarcinogenic action of curcumin in liver, even after withdrawal of treatment in Dalton's lymphoma bearing mice. Oxidative stress observed during lymphoma progression reduced antioxidant enzyme activities, and induced angiogenesis as well as activation of early stress activated genes and glycolytic pathway. Curcumin treatment resulted in activation of antioxidant enzyme super oxide dismutase and down regulation of ROS level as well as activity of ROS producing enzyme NADPH:oxidase, expression of stress activated genes HIF-1α, cMyc and LDH activity towards normal level. Further, it lead to significant inhibition of angiogenesis, observed via MMPs activity, PKCα and VEGF level, as well as by matrigel plug assay. Thus findings of this study conclude that the long term effect of curcumin shows anticarcinogenic potential via induction of antioxidant defense system and inhibition of angiogenesis via down regulation of stress activated genes and glycolytic pathway in liver of lymphoma bearing mice.

## Introduction

Elevated reactive oxygen species (ROS) such as superoxide radicals and H_2_O_2_ function as signaling molecules in various aspects of growth factor-mediated responses including angiogenesis during cancer progression. The major source of ROS production is regulated by NADPH oxidase. The vascular endothelial growth factor (VEGF) is a major angiogenesis inducer, which stimulates proliferation, migration, and tube formation of endothelial cells (ECs) through the VEGF receptor type2. VEGF induced angiogenesis is mediated by protein kinase C (PKC), as PKC is involved in the signaling pathway of VEGF-mediated tumor development and angiogenesis [1]. PKCα promotes angiogenic activity of endothelial cells via induction of VEGF and VEGF enhances its own expression via PKCα-dependent positive feedback mechanism [2]. Further, VEGF is regulated at transcriptional level by hypoxia-inducible factor 1-α (HIF-1α) in response to hypoxia [3], [4]. HIF-1 not only regulates oxygen delivery (angiogenesis), but oxygen consumption (glycolytic metabolism) also in hypoxic tumor microenvironment [5]. Expression of a number of genes starting with oncogene-mediated followed by HIF-1 mediated genes are induced that promote metabolic changes during carcinogenesis. It results in the highly glycolytic “Warburg” phenotype and suppression of mitochondrial biogenesis [6]. The upregulation of the lactate dehydrogenase A (LDH-A) is a major molecular mediator of Warburg effect, which occurs even in aerobic condition as a consequence of hypoxic tumour microenvironment and alterations in certain oncogenes or tumor suppressor genes [7]. In addition, the overexpression of LDH-A is regulated by HIF-1 cooperation with deregulated c-Myc. It has been observed that oncogenic activation through deregulated expression of c-Myc contributes to tumorigenesis in various tissues in transgenic mice and many types of human cancers including lymphomas [8]. Thus, deregulation in expression of oncogenes, tumor-suppressor genes and stability genes are involved in carcinogenesis, that result in a reduced level of antioxidant defense in tumor microenvironment and cancer cells [9]. Further, matrix metalloproteinase (MMPs) are zinc dependent proteolytic enzymes, cleave extracellular matrix as well as non-matrix substrates (growth factors, cell surface receptors, etc) and regulate signaling pathways that control cell growth, inflammation, or angiogenesis. It acts as modulator of the tumor microenvironment and may even work in a nonproteolytic manner. The deregulation of MMPs is involved in many diseases, such as tumor metastasis, rheumatoid arthritis, and periodontal disease [10], [11]. Besides, MMPs reactivate angiogenic activity of VEGF by selective degradation of connective tissue growth factor (CTGF), as one of the isoforms of VEGF binds CTGF and angiogenic activity of VEGF is inhibited in the VEGF-CTGF complex [12]. Thus, inhibition of angiogenic activities via modulation of glycolytic pathway, activation of endogenous antioxidant defense system and inhibition of ROS formation might be a step to prevent carcinogenesis. During progression of Dalton's lymphoma (a murine transplantable non-Hodgkin's T-cell lymphoma), different parts are affected including liver and bone marrow beyond the lymphatic system. Liver being the major metabolic and detoxifying organ of the body is greatly affected during lymphoma progression. In addition, metastasis and malignancy has been confirmed earlier in liver of Dalton's lymphoma bearing (DL) mice, which are characteristics of non-Hodgkin's lymphoma [13], [14].

Broad array of naturally occurring phytochemicals may confer optimal health benefits for humans. The use of whole extract or single-isolated constituent or a metabolite of an isolated constituent to achieve desirable health benefits has been an issue of the past several years. Curcumin, a naturally occurring chemopreventive phytochemical derived from turmeric (*Curcuma longa*), is reported to inhibit chemically induced carcinogenesis in multiple organ sites in various experimental animal models. Curcumin suppresses activation of several transcription factors that are implicated in oncogenic activities, blocks transformation, proliferation, and invasion as well as induces apoptosis in tumor cells. Thus curcumin shows immense promise for treatment of cancer [15]. However it is still to be reported whether the anticarcinogenic action of curcumin is due to the cumulative effect of its metabolites or due to curcumin itself, as metabolism of curcumin is very fast but metabolites remain for longer time in different tissues [16]. Further, accumulating evidences suggest that the consumption of whole or partially purified food extracts is more beneficial over single-isolated constituent due to the existence of synergistic interactions among phytochemicals in whole foods [17], [18]. Therefore, present study was designed to investigate the mechanism involved in anticarcinogenic action of curcumin even after withdrawal of administration. The study is focused on glycolytic pathway and angiogenesis, cooperatively regulated by c-Myc and HIF-1 under oxidative tumour microenvironment in metastatic liver of lymphoma bearing mice.

## Materials and Methods

### Reagents

All chemicals were of analytical and molecular biology grade as well as endotoxin free and used without further purification. Curcumin, reagents for RNA isolation and Taq polymerase, Nonfluorescent 2′, 7′-dichlorofluorescein diacetate (H2DCFDA) and HRP conjugated anti-β actin were purchased from Sigma Aldrich. Reverse Transcriptase, Ribonuclease Inhibitor, random primers and 100 bp Plus DNA ladder from Fermentase Life Science and gene specific primers for RT-PCR were synthesized from Metabion. Anti-mouse PKCα and VEGF-A antibody were purchased from Santacruz biotechnology and Biolegend respectively. HRP conjugated goat anti-rabbit and rabbit anti-rat secondary antibody from Bangalore Genei. Matrigel basement membrane matrix from BD Biosciences, gelatin from amresco and ECL (Super signal Kit) was purchased from PIERCE Biotechnology.

### Animals and induction T-cell Lymphoma (Dalton's Lymphoma)

Mice (AKR strain) were bred and maintained under standard laboratory conditions with proper human care, as per the guidelines of the institutional animal ethical committee, at 25±2°C under a 12 h light/12 h dark schedule provided with standard mice feed and drinking water *ad libitum*. All the animal experiments were performed with the approval of the Institutional Animal Ethical Committee, Banaras Hindu University. Healthy adult male mice (16–20 weeks old and 30±2 g) were used in the experimental work. Dalton's lymphoma (transplantable non-Hodgkin's T-cell lymphoma) ascite cells were transplanted to adult male mice through intraperitoneal (i.p.) serial transplantation of about 1×10^6^ viable ascite tumor cells in 1 ml of phosphate buffer saline (PBS) per mouse as described earlier [19]. Dalton's lymphoma ascite cells were gifted by Prof. Ajit Sodhi, School of Biotechnology, Banaras Hindu University, Varanasi, India. Dalton's lymphoma is a transplantable murine T-cell lymphoma originated in the thymus gland of a DBA/2 mouse at the National Cancer Institute, Bethesda, M D, in 1947. [20].

Development of DL was confirmed by abnormal abdominal swelling and increased body weight, which were visible clearly on 10–11 days post transplantation and DL mice survived for 20±2 days. First 7–9 days starting from next day of DL transplantation, growth of Dalton's lymphoma did not show any major change in body weight and ascite fluid accumulation. Thus first 7–9 days can be compared with the lag-phase/preparatory-phase of sigmoid curve for ascite cell population growth. Therefore, schedule of curcumin treatment to DL mice was selected accordingly for 9 days starting from next day after DL transplantation and mice were sacrificed on day 18 of post DL transplantation (before DL mice dies naturally) to get the long term effect of curcumin, as curcumin must be completely metabolized to its metabolites before 9 days from last day of treatment. Hence, the effect would be not due to the direct effect of curcumin but might be due to the metabolites of curcumin.

### Schedule of curcumin treatment to DL mice and tissue collection

One group of normal mice was used as control, without any treatment (N). DL mice were randomly divided into five groups with 6 mice in each group (n = 6). Three groups were treated with different doses of curcumin: 1.5 mg [Dalton's lymphoma bearing mice treated with 50 mg curcumin/kg body weight (DLT50)], 3 mg [Dalton's lymphoma bearing mice treated with 100 mg curcumin/kg body weight (DLT100)], and 4.5 mg [Dalton's lymphoma bearing mice treated with 150 mg curcumin/kg body weight (DLT150)], dissolved in 50 µl of DMSO to each mouse per day through i.p. for 9 days consecutively, starting from the next day after DL transplantation. One group of DL mice received 50 µl DMSO as vehicle (DL+DMSO) in similar manner and other group of DL mice were used without any treatment (DL). All the mice from each group were sacrificed on day 18 of post DL transplantation by cervical dislocation. Liver was excised immediately after sacrificing the animal and washed in chilled normal saline. Liver of all animals (n = 6) of one group was pooled and mixed by chopping aseptically at 4°C for average result. Collected tissue was used immediately or preserved at −80°C for further study.

### *In vivo* angiogenesis assay

Another set of six groups with three mice per groups were taken to study *in vivo* angiogenesis. All the treatments were same as above except that, at the time of DL transplantation, each mice from all the groups were subcutaneously injected with 500 µl of BD Matrigel basement membrane matrix (Matrigel plug). Just after sacrificing, matrigel plugs were removed. Plugs were immediately photographed and weighed. To quantitate the vascularisation of the plug, the amount of hemoglobin (Hb) accumulated in the plug was measured using HEMOCOR-D kit (Crest Biosystems, Tulip Group, India) following the manufacturer's protocol and absorbance of samples were measured at 540 nm.

### RT-PCR

Total RNA was isolated using TRI Reagent (Sigma-Aldrich) as per its user manual. DNase treatment was carried out on total RNA using TURBO DNA-Free™ Kit I to remove any genomic DNA contamination. RNA was quantified at 260 nm and the integrity was checked by 1% formaldehyde agarose gel electrophoresis. The cDNA was synthesized from isolated total RNA using a standard mixture containing each dNTPs, random hexamer, M-MuLV reverse transcriptase, RNase inhibitor and reaction buffer according to the standard protocol of Fermentas Life Science, and cDNA was used immediately or stored at −80°C. Expression of isozymes of SOD and NOX, HIF-1α, cMyc, LDH-A, PKCα, VEGF-A and MMP 9 & 2 genes were studied by semi-quantitative RT-PCR using synthesized cDNA. Taq polymerase and appropriate primer pairs (Table-1) were used for PCR reactions using Thermal cycler (Applied Biosystem). PCR was started with 3 min at 95°C for denaturation followed by three-step temperature cycle (Table-1). A final extension step at 72°C for 7 min was included after the final cycle to complete polymerization. Number of cycles was optimized within the exponential phase of amplification. The size of amplified products was checked by agarose gel electrophoresis using 100 bp ladder. The band intensity of amplified products in the agarose gel was visualized, photographed and analyzed by using Gel Doc System (Alpha Innotech^EC^). Further, band intensity was normalized with corresponding lane of β-actin as internal control.

**Table 1:** Primer pairs and conditions of PCR.

Genes with accession no.	Sequences of primer pairs (F: forward, R: reverse)	PCR condition (annealing and elongation)	No. of cycles	Amplicon size (bp)
Cu/Zn-SOD	F: 5′-ATCCACTTCGAGCAGAAG-3′	55°C-30 sec	25	340
(NM_011434.1)	R: 5′-TTCCACCTTTGCCCAAGT-3′	72°C-45 sec		
Mn-SOD	F: 5′-AGCGGTCGTGTAAACCTCA-3′	55°C-30 sec	28	439
(NM_013671.3)	R: 5′-AGACATGGCTGTCAGCTTC-3′	72°C-45 sec		
NOX1	F: 5′-CCAGCGTGCCGACAACAAGC-3′	63°C-30 sec	32	374
(XM_006528515.1)	R: 5′-GCTGACAGCGTTTGCGCAGG-3′	72°C-40 sec		
NOX2	F: 5′-AGGGGTTCCAGTGCGTGTTGC-3′	63°C-45 sec	28	278
(XM_006527565.1)	R: 5′-GTCACGGCCACATACAGGCCC-3′	72°C-45 sec		
HIF-1α	F: 5′-AGCCCTAGATGGCTTTGTGA-3′	57°C-45 sec	25	467
(NM_010431.2)	R: 5′-TATCGAGGCTGTGTCGACTG-3′	72°C-45 sec		
cMyc	F: 5′-TTCTCAGCCGCTGCCAAGCTGGTC-3′	64°C-30 sec	28	455
(NM_001177353.1)	R: 5′-GGTTTGCTGTGGCCTCGGGATGGA-3′	72°C-30 sec		
LDH-A	F: 5′-ATGCACCCGCCTAAGGTTCTT-3′	55°C-30 sec	28	103
(NM_010699.2)	R: 5′-TGCCTACGAGGTGATCAAGCT-3′	72°C-30 sec		
PKC-α	F: 5′-GGTTTGGGAAACAAGGCTTC-3′	58°C-45 sec	28	278
(NM_011101.3)	R: 5′- GCAGAGGCTAGGGACATTGA-3′	72°C-45 sec		
VEGF-A	F: 5′-ACCAGCCCGGGAGTCTGTGC-3′	65°C-30 sec	30	470
(NM_001025250.3)	R: 5′-CTTCGGTTCCTCGCGGCTCG-3′	72°C-40 sec		
MMP9	F: 5′-CGTGTACGGACCCGAAGCGG-3′	61°C-45 sec	26	299
(NM_013599.3)	R: 5′-TGCTACACCAAGGCGTGCCG-3′	72°C-45 sec		
MMP2	F: 5′-CGTTCCGCTTCCAGGGCACC-3′	61°C-45 sec	27	239
(NM_008610.2)	R: 5′-CACCTTGCCATCGTTGCGGC-3′	72°C-45 sec		
β-actin	F: 5′-GTGGGCCGCCCTAGGCACCAG-3′	60°C-45 sec	26	539
(NM_007393.3)	R: 5′-TCTTTGATGTCACGCACGATTTC-3′	72°C-45 sec		

### Non-denaturing PAGE and specific activity staining

Activity gel assays were used to measure the enzyme activity and isozymes pattern of antioxidant enzyme SOD as well as NOX and LDH. As alterations of enzymatic activity is associated with metabolic changes, non-denaturing PAGE analysis and activity gel assay was preferred over immunodetection, because, the method utilizes substrate specificity based detection of only active part of protein. Thus, it is considered highly relevant for correlating a change in the level of a specific isozyme with that of metabolic alterations at cellular level.

### Superoxide dismutase (SOD)

The activity gel assay of SOD was performed by non-denaturing PAGE, followed by activity staining as per the method of Beauchamp and Fridovich [21]. Equal amount of protein from each sample was separated by 10% non-denaturing PAGE at 4°C. After electrophoresis the gel was soaked in 1.23 mM NBT solution for 20 min under dark. The gel was briefly washed in distilled water and incubated for 15–20 min under dark in 100 mM phosphate buffer (pH-7.0) containing 28 mM TEMED and 0.28 mM riboflavin. Then the gel was exposed to a fluorescent light until appearance of clear zones of aromatic activity bands with blue background. The intensity of bands was analyzed by densitometric scanning using an Alpha Image Analyser System (Alpha Innotech, San Leandro, CA, U.S.A.).

### Activity staining of NADPH: Oxidase

The level of enzymatic activity of NOX isozymes were identified in non-denaturing PAGE, followed by activity staining by NBT reduction method described by Sagi and Fluhr [22]. Equal amount of protein from each sample was separated by 10% non-denaturing PAGE at 4°C. After electrophoresis the gel was stained in 50 mM Tris-Cl (pH-7.4) 0.2 mM NBT, 0.1 mM MgCl2, and 1 mM CaCl2, in the dark for 20 min. Then 0.2 mM NADPH was added to the staining solution and the appearance of blue formazan bands was observed. The reaction was stopped by immersion of the gels in distilled water. The intensity of bands was analyzed by densitometric scanning using an Alpha Image Analyser System (Alpha Innotech, San Leandro, CA, U.S.A.).

### Activity staining of Lactate Dehydrogenase (LDH)

In gel enzymatic activity assay of lactate dehydrogenase was performed by non-denaturing PAGE followed by specific activity staining as described by Dietz and Lubrano [23]. Equal amount of protein from each sample was separated by 8% non-denaturing PAGE at 4°C. After electrophoresis the gel was subjected to LDH specific activity staining in the staining solution containing 125 mM Tris-Cl (pH-7.4), 0.5 mM magnesium chloride, 0.1 mM lithium-lactate, 1 mg/ml NAD, 10 mM NaCl, 0.25 mg/ml NBT and 0.025 mg/ml PMS with gentle shaking for 5–10 min at RT, after development of LDH bands the gel was washed. The intensity of bands was analyzed by densitometric scanning using an Alpha Image Analyser System (Alpha Innotech, San Leandro, CA, U.S.A.).

### Hydrogen peroxide assay

Hydrogen peroxide level in the liver tissue was measured spectrophotometrically by taking the absorbance at 570 nm as described previously [24]. The concentration of H_2_O_2_ in each sample was determined using the standard curve, generated by taking known quantities of H_2_O_2_, and expressed in µmole/mg protein.

### ROS Assay

Total ROS level was determined by the oxidative conversion of nonfluorescent 2′, 7′-dichlorofluorescein diacetate (H2DCFDA) to highly fluorescent 2′, 7′-dichlorofluorescein (DCF) as described previously [25]. Liver extracts of amount 100 µl were incubated at 37°C for 60 min with 100 µl of 2 mM H2DCFDA (Invitrogen) in PBS. Fluorescence was recorded at 485 nm (excitation) and 527 nm (emission) with HITACHI F-3000 fluorescence spectrophotometer. The level of ROS in each sample was determined by observing fluorescence (absorbance)/mg protein.

### Gelatin Zymography

Activity of MMPs in tissue sample was analyzed to study gelatin degrading activity using zymography as described previously [26]. Equal amount of protein from each sample mixed with equal volume of 2X non-reducing buffer (0.125 M Tris-Cl pH-6.8, 20% glycerol, 4% SDS, 0.003% bromophenol blue) was separated at 4°C by 8% resolving and 5% stacking SDS-PAGE containing 0.1% gelatin. Following electrophoresis, gel was washed twice in 2.5% Triton X-100 for 20 min each in order to remove SDS and renature enzymes. The gel was then incubated for 20 hour at 37°C in the buffer containing 21 mM Tris-Cl (pH-7.6), 10 mM CaCl2, and 0.04% NaN3. Then gel was washed in distilled water, stained with CBB and destained in methanol and acetic acid. Protease activity was observed as a clear band of digested gelatin. The intensity of bands was analyzed by densitometric scanning using an Alpha Image Analyser System (Alpha Innotech, San Leandro, CA, U.S.A.).

### Statistical analysis

Statistical analysis was performed by SPSS software using one-way ANOVA followed by Tucky's test. Values were expressed as mean ± S.E.M. obtained from three different sets of experiments, p<0.05 was taken as statistically significant (95% confidence interval). #p<0.05 and ##<0.01 compared to N, *p<0.05 and **p<0.01 compared DL+DMSO group respectively.

## Results

### Expression and enzymatic activity of SOD

**Figure 1:**
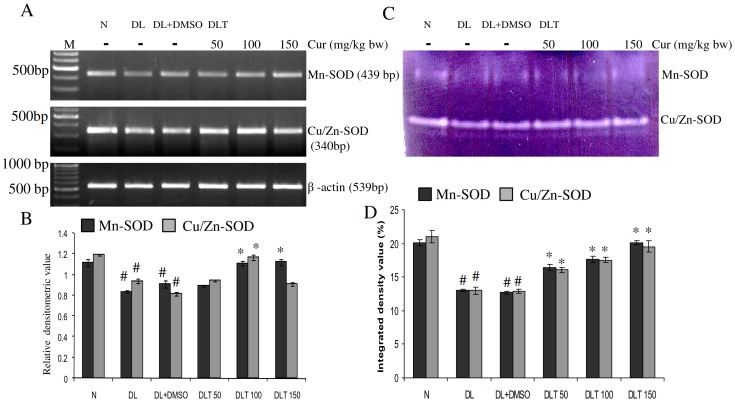
Expression and activities of SOD isozymes. Effect of curcumin on mRNA expression and enzymatic activity of SOD isozymes in liver of lymphoma bearing mice (A) RT-PCR of Mn-SOD, Cu/Zn-SOD and β-actin gene, (B) Densitometric scanning of Mn-SOD and Cu/Zn-SOD genes after normalization with β-actin, (C) Specific staining showing activity of SOD isozymes, (D) Densitometric scanning of the activity band of SOD isozymes. Livers of all six animals of each group was pooled separately and used for extraction of total RNA and proteins at non denaturing condition. Data represent mean ± S.E.M. #p<0.05 compared to N group and *p<0.05 compared to DL+DMSO group respectively. Cur is curcumin, M is 100 bp marker and bw is body weight. N, DL, DL+DMSO, DLT50, DLT100 and DLT150 represents normal, Dalton's lymphoma bearing, Dalton's lymphoma bearing mice treated with DMSO and Dalton's lymphoma bearing mice treated with 50, 100 and 150 mg curcumin/kg body weight dissolved in DMSO respectively.

The expression of SOD in liver of DL and DL+DMSO mice was found to be down regulated significantly compared to normal. The expressions of both Mn-SOD and Cu/Zn-SOD in DL mice were 75% and 78% of normal mice respectively and 82% and 69% of normal respectively in DL+DMSO. Significant up regulation of the expression of both the isozymes of SOD was found towards normal level by curcumin treatment; Mn-SOD was upregulated upto 1.21 fold and 1.22 fold of DL+DMSO mice with 100 and 150 mg curcumin/kg bw respectively and Cu/Zn-SOD upto 1.15 fold, 1.42 fold and 1.1 fold of DL+DMSO with 50, 100 and 150 mg curcumin/kg bw respectively [Fig. 1(A)]. Similarly activity of SOD also follows the similar trend of variation in expression. The activity of Mn-SOD in DL and DL+DMSO mice was found to be 65% and 63% of normal mice respectively. Curcumin treatment elevated the activity in dose dependent manner which was approximately upto 1.3 fold, 1.39 fold and 1.58 fold of DL+DMSO in 50, 100 and 150 mg curcumin/kg bw respectively. Similarly, the activity of Cu/Zn-SOD was decreased in DL and DL+DMSO mice upto 61% and 61.5% of normal mice respectively. Curcumin treatment was capable of increasing the activity of Cu/Zn-SOD in similar dose dependent manner, approximately upto 1.24 fold, 1.35 fold, 1.5 fold of DL+DMSO mice with 50, 100 and 150 mg curcumin/kg bw respectively [Fig. 1(C)].

### Expression and enzymatic activity of NOX

**Figure 2:**
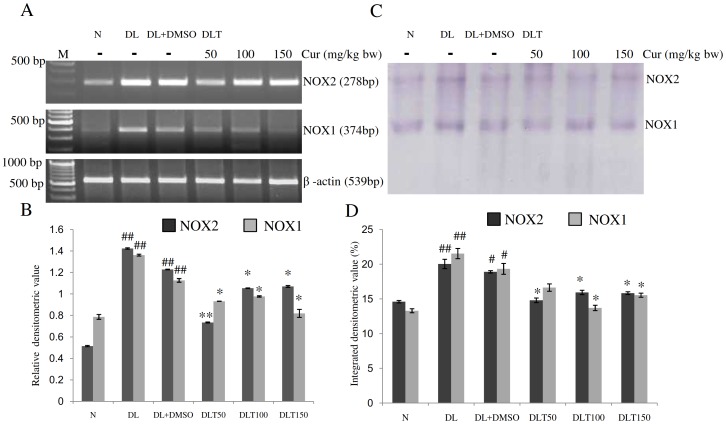
Expression and activities of NOX isozymes. Effect of curcumin on mRNA expression and enzymatic activity of NOX isozymes in liver of lymphoma bearing mice (A) RT-PCR of NOX2, NOX1 and β-actin genes, (B) Densitometric scanning of NOX2 and NOX1 genes after normalization with β-actin, (C) Specific staining showing activity of NOX isozymes, (D) Densitometric scanning of the activity band of NOX isozymes. Livers of all six animals of each group were pooled separately and used for extraction of total RNA and proteins at non denaturing condition. Data represent mean ± S.E.M. #p<0.05 and ##<0.01 compared to N group, *p<0.05 and **p<0.01 compared to DL+DMSO group respectively. Cur is curcumin, M is 100 bp marker and bw is body weight. N, DL, DL+DMSO, DLT50, DLT100 and DLT150 represents normal, Dalton's lymphoma bearing, Dalton's lymphoma bearing mice treated with DMSO and Dalton's lymphoma bearing mice treated with 50, 100 and 150 mg curcumin/kg body weight dissolved in DMSO respectively.

The expression of NOX1 and NOX2 was observed to be significantly upregulated in DL and DL+DMSO mice as compared to normal mice, which was modulated towards normal level by curcumin treatment [Fig. 2(A)]. The expression of NOX1 was found to be approximately 1.75-fold and 1.44-fold of normal mice in the liver of DL and DL+DMSO mice respectively. Curcumin treatment reduced the expression which was approximately 83%, 86% and 72% of DL+DMSO mice with the dose of 50, 100 and 150 mg/kg bw respectively. Similarly compared to normal, expression of NOX2 was found to be approximately 2.7-fold and 2.38-fold in the liver of DL and DL+DMSO mice respectively, which was down regulated after curcumin treatment as observed to be approximately 60%, 86% and 87% of DL+DMSO mice with the dose of 50, 100 and 150 mg/kg bw respectively. Similarly activity of NOX was observed to be elevated as its mRNA expression. NOX regulates the formation of ROS; therefore activity of NOX can be measured as an indicator of ROS level. Compared to normal mice, activity of NOX1 was approximately 1.6-fold and 1.45-fold where as activity of NOX2 was 1.37-fold and 1.3-fold in the liver of DL and DL+DMSO mice respectively. Different doses of curcumin treatment significantly modulated the activity towards normal level. Activity of NOX1 was found to be approximately 86%, 70% and 80% of DL+DMSO mice, while activity of NOX2 was found to be approximately 78%, 84% and 84% of DL+DMSO mice with the dose of 50, 100 and 150 mg/kg bw respectively [Fig. 2(C)].

### Level of H_2_O_2_ and total ROS in liver tissue

**Figure 3:**
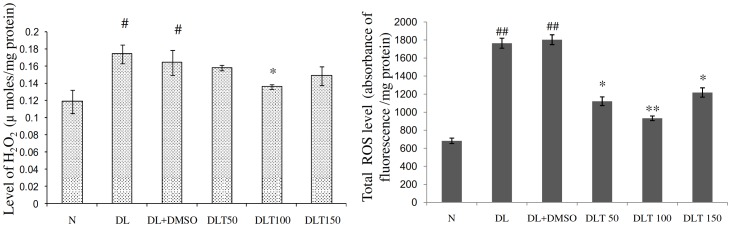
Total H_2_O_2_ and ROS level. Effect of curcumin on oxidative stress in terms of total H2O2 level and total ROS level in liver of lymphoma bearing mice (A) Histogram shows total H2O2 level in different groups, (B) Histogram shows total ROS level in different groups. Livers of all six animals of each group were pooled separately and homogenate was prepared for determination of H2O2 and ROS level. Data represent mean ± S.E.M. #p<0.05 compared to N group, *p<0.05 compared to DL+DMSO group respectively. N, DL, DL+DMSO, DLT50, DLT100 and DLT150 represents normal, Dalton's lymphoma bearing, Dalton's lymphoma bearing mice treated with DMSO and Dalton's lymphoma bearing mice treated with 50, 100 and 150 mg curcumin/kg body weight dissolved in DMSO respectively.

The level of H2O2, being an indicator of oxidative stress reflects the oxidative status of tissue. The level of hydrogen peroxide in the liver of DL and DL+DMSO mice was elevated significantly, upto approximately 1.47 fold and 1.39 fold of normal mice respectively. Curcumin treatment significantly reduced the level upto 82% of DL+DMSO mice with 100 mg curcumin/kg bw mice, and 96% and 90% of DL+DMSO mice with 50 and 150 mg curcumin/kg bw mice respectively [Fig. 3(A)]. ROS synthesized by NOX as well from other sources acts as an important signaling molecule in oxidative tumor microenvironment to induce oncogenic transformation. Total ROS level in terms of fluorescence (absorbance)/mg protein was significantly elevated in DL and DL+DMSO mice upto approximately 2.59-fold and 2.64-fold of normal mice. Treatment of curcumin suppressed ROS level significantly, which was observed to be approximately 62%, 51% and 67% of DL+DMSO mice with the dose of 50, 100 and 150 mg/kg bw respectively [Fig. 3(B)].

### Expression of HIF-1α and cMyc

**Figure 4:**
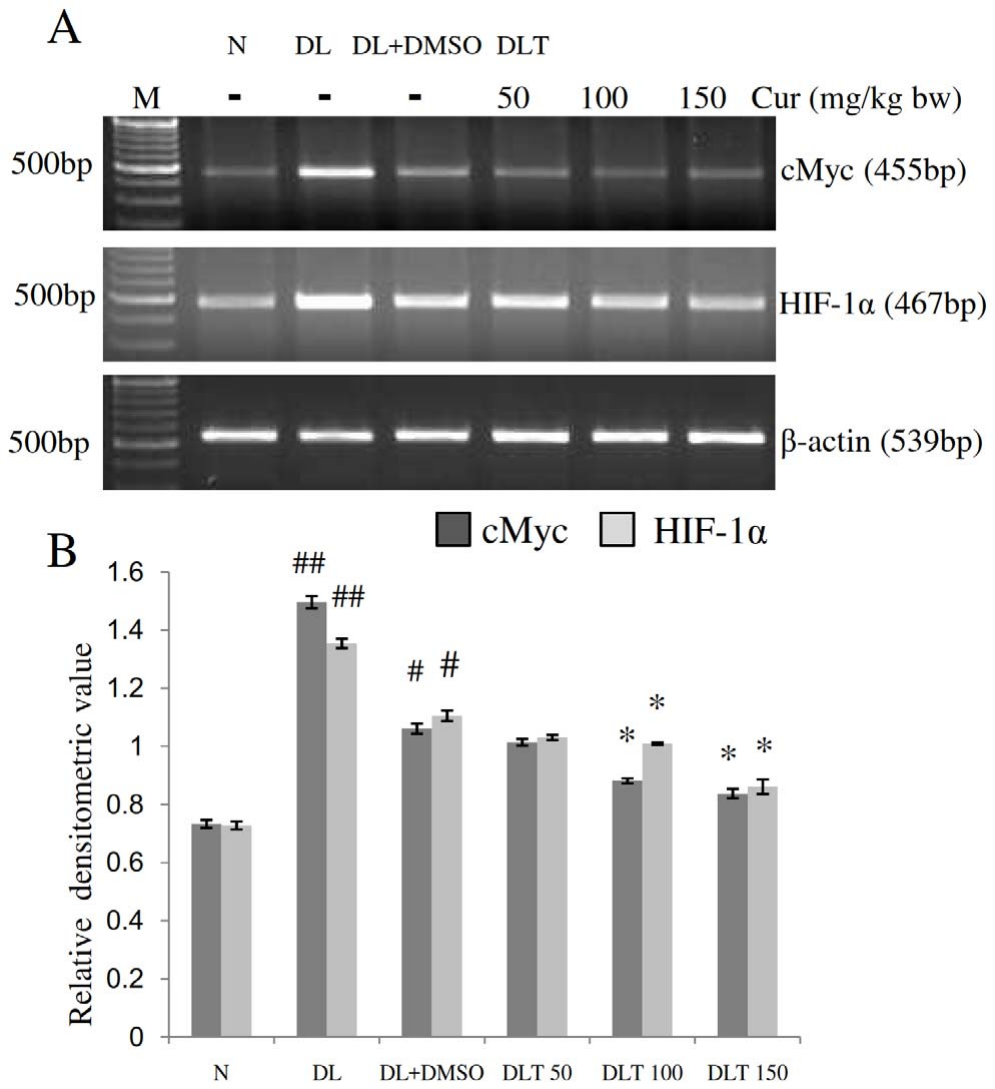
Expression of cMyc and HIF-1α. Effect of curcumin on mRNA expression of stress activated gene HIF-1α and cMyc in liver of lymphoma bearing mice (A) RT-PCR of HIF-1α, cMyc and β-actin genes, (B) Densitometric scanning of HIF-1α and cMyc genes after normalization with β-actin. Livers of all six animals of each group were pooled separately and used for extraction of total RNA. Data represent mean ± S.E.M. #p<0.05 and ##<0.01 compared to N group, *p<0.05 compared to DL+DMSO group respectively. Cur is curcumin, M is 100 bp marker and bw is body weight. N, DL, DL+DMSO, DLT50, DLT100 and DLT150 represents normal, Dalton's lymphoma bearing, Dalton's lymphoma bearing mice treated with DMSO and Dalton's lymphoma bearing mice treated with 50, 100 and 150 mg curcumin/kg body weight dissolved in DMSO respectively.

The mRNA expression of HIF-1α and cMyc was studied as an early response stress gene and oncogene respectively in hypoxic tumor microenvironment. The expression of both HIF-1α and cMyc was up regulated in DL and DL+DMSO mice as compared to normal mice, which was significantly lowered by curcumin treatment [Fig. 4(A)]. The expression of HIF-1α in liver of DL and DL+DMSO mice was found to be approximately 1.85-fold and 1.51-fold of normal mice respectively. Expression level after curcumin treatment was approximately 93%, 91% and 78% of DL+DMSO mice with the dose of 50, 100 and 150 mg/kg bw respectively. Similarly, expression of cMyc was found to be approximately 2.03-fold and 1.45-fold of normal mice in the liver of DL and DL+DMSO mice respectively and after curcumin treatment the expression was decreased to approximately 95%, 83% and 78% of DL+DMSO mice with the dose of 50, 100 and 150 mg/kg bw respectively.

### Expression and enzymatic activity of LDH-A

**Figure 5:**
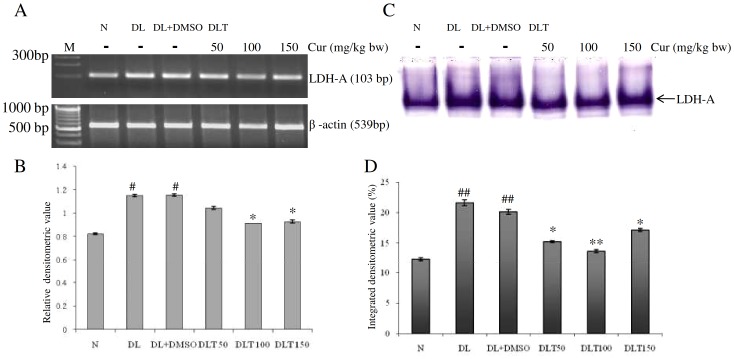
Expression and activity of LDH. Effect of curcumin on mRNA expression and enzymatic activity of LDH-A in liver of lymphoma bearing mice (A) RT-PCR of LDH-A and β-actin genes, (B) Densitometric scanning of LDH-A after normalization with β-actin, (C) Specific staining showing activity of LDH-A, (D) Densitometric scanning of the activity band of LDH-A. Livers of all six animals of each group were pooled separately and used for extraction of total RNA and total proteins at non denaturing condition. Data represent mean ± S.E.M. #p<0.05 and ##<0.01 compared to N group, *p<0.05 and **p<0.01 compared to DL+DMSO group respectively. Cur is curcumin, M is 100 bp marker and bw is body weight. N, DL, DL+DMSO, DLT50, DLT100 and DLT150 represents normal, Dalton's lymphoma bearing, Dalton's lymphoma bearing mice treated with DMSO and Dalton's lymphoma bearing mice treated with 50, 100 and 150 mg curcumin/kg body weight dissolved in DMSO respectively.

The mRNA expression of LDH-A in liver of DL and DL+DMSO mice was upregulated up to approximately 1.4-fold and 1.4-fold of normal mice respectively. All the doses of curcumin significantly down regulated the expression. The expression after curcumin treatment was found to be approximately 90%, 79% and 80% of DL+DMSO mice with the dose of 50, 100 and 150 mg/kg bw respectively [Fig. 5(A)]. Pyruvate cannot be metabolized aerobically but is converted into lactate by LDH-A in hypoxic tumor microenvironment. Therefore, glycolytic metabolism can be monitored in terms of the activity of LDH-A. The activity of LDH-A was found to be elevated upto approximately 1.76-fold and 1.64-fold of normal mice in the liver of DL and DL+DMSO mice respectively. Treatment of curcumin significantly reduced the activity of LDH-A, which was approximately 75%, 67% and 85% of DL+DMSO mice with the doses of 50, 100 and 150 mg/kg bw respectively [Fig. 5(C)].

### Expression and protein level of PKCα

**Figure 6:**
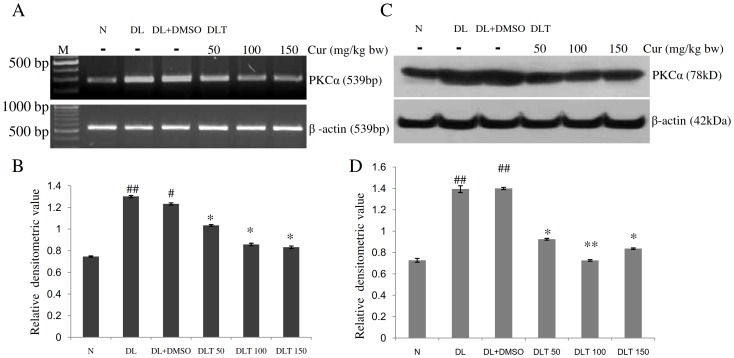
Expression of PKCα. Effect of curcumin on mRNA expression and protein level of PKCα in liver of lymphoma bearing mice (A) RT-PCR of PKCα and β-actin genes, (B) Densitometric scanning of PKCα after normalization with β-actin, (C) Protein level of PKCα and β-actin analysed by Western blot analysis, (D) Densitometric scanning of PKCα after normalization with β-actin. Livers of all six animals of each group were pooled separately and used for extraction of total RNA and proteins. Data represent mean ± S.E.M. #p<0.05 and ##<0.01 compared to N group, *p<0.05 and **p<0.01 compared to DL+DMSO group respectively. Cur is curcumin, M is 100 bp marker and bw is body weight. N, DL, DL+DMSO, DLT50, DLT100 and DLT150 represents normal, Dalton's lymphoma bearing, Dalton's lymphoma bearing mice treated with DMSO and Dalton's lymphoma bearing mice treated with 50, 100 and 150 mg curcumin/kg body weight dissolved in DMSO respectively.

The mRNA expression of PKCα was found to be up regulated in DL and DL+DMSO mice upto approximately 1.75-fold and 1.65-fold of normal mice. All the three doses of curcumin reduced the expression of PKCα significantly. Down regulation of the expression of PKCα was upto approximately 83%, 69% and 67% of DL+DMSO mice with 50, 100 and 150 mg curcumin/kg bw respectively (Fig. 6(A)]. Protein level of PKCα follows the similar variation pattern of its mRNA expression. In case of DL and DL+DMSO mice the protein level was found to be approximately 1.9 fold and 1.92 fold of normal mice respectively. Treatment of curcumin resulted in significant decrease of the level of PKCα. The protein level was approximately 66%, 52% and 59% of DL+DMSO mice with 50, 100 and 150 mg curcumin/kg bw respectively [Fig. 6(C)].

### Expression and protein level of VEGF-A

**Figure 7:**
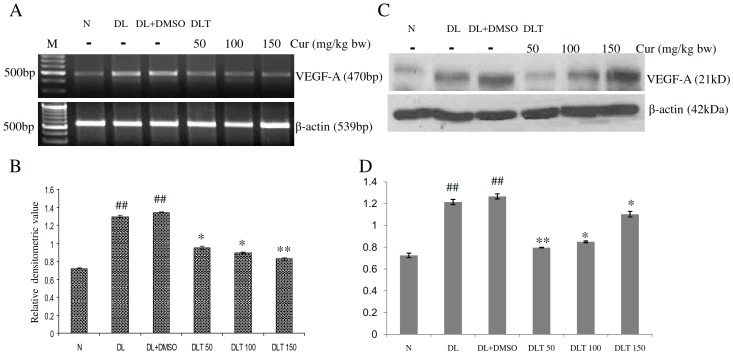
Expression of VEGF-A. Effect of curcumin on mRNA expression and protein level of VEGF-A in liver of lymphoma bearing mice (A) RT-PCR of VEGF-A and β-actin genes, (B) Densitometric scanning of VEGF-A after normalization with β-actin, (C) Protein level of VEGF-A and β-actin analysed by Western blot analysis, (D) Densitometric scanning of VEGF-A after normalization with β-actin. Livers of all six animals of each group were pooled separately and used for extraction of total RNA and proteins. Data represent mean ± S.E.M. #p<0.05 and ##<0.01 compared to N group, *p<0.05 and **p<0.01 compared to DL+DMSO group respectively. Cur is curcumin, M is 100 bp marker and bw is body weight. N, DL, DL+DMSO, DLT50, DLT100 and DLT150 represents normal, Dalton's lymphoma bearing, Dalton's lymphoma bearing mice treated with DMSO and Dalton's lymphoma bearing mice treated with 50, 100 and 150 mg curcumin/kg body weight dissolved in DMSO respectively.

The mRNA expression of VEGF-A was upregulated in the liver of DL and DL+DMSO mice, which was approximately 1.78-fold and 1.89-fold of normal mice respectively. Curcumin treatment modulated the expression towards normal level by reducing it in a dose dependent manner. The expression was approximately 70%, 65% and 62% of DL+DMSO mice with the dose of 50, 100 and 150 mg/kg bw respectively [Fig. 7(A)]. Similarly protein level of VEGF-A in case of DL and DL+DMSO mice was found to be approximately 1.68-fold and 1.75-fold of normal mice. Curcumin treatment significantly reduced the level of VEGF-A towards normal. The protein level was observed to be approximately 63%, 67% and 87% of DL+DMSO mice with the doses of 50, 100 and 150 mg/kg bw treated groups respectively [Fig. 7(C)].

### Expression and protease activity of MMP-9 and MMP-2

**Figure 8:**
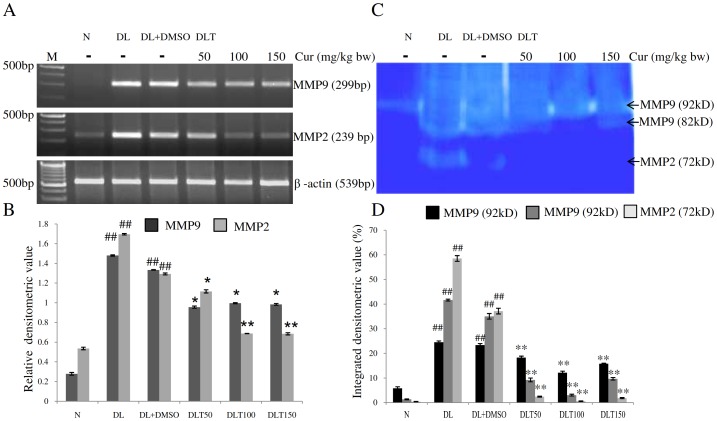
Expression and activity of MMPs. Effect of curcumin on mRNA expression and protease activity of MMP 9 and 2 in liver of lymphoma bearing mice (A) RT-PCR of MMP9, MMP2 and β-actin genes, (B) Densitometric scanning of MMP9 and MMP2 genes after normalization with β-actin, (C) Gelatin Zymography showing protease activity of MMP9 and MMP2, (D) Densitometric scanning of the activity band of MMP9 and MMP2. Livers of all six animals of each group were pooled separately and used for extraction of total RNA and proteins at non denaturing condition. Data represent mean ± S.E.M. #p<0.05 and ##<0.01 compared to N group, *p<0.05 and **p<0.01 compared to DL+DMSO group respectively. Cur is curcumin, M is 100 bp marker and bw is body weight. N, DL, DL+DMSO, DLT50, DLT100 and DLT150 represents normal, Dalton's lymphoma bearing, Dalton's lymphoma bearing mice treated with DMSO and Dalton's lymphoma bearing mice treated with 50, 100 and 150 mg curcumin/kg body weight dissolved in DMSO respectively.

The mRNA expression of MMP-9 and MMP-2 was studied as an inducer of angiogenesis in the tumor microenvironment of metastatic liver of lymphoma bearing mice. The expression of both MMP-9 and MMP-2 was significantly upregulated in DL and DL+DMSO mice as compared to normal mice, which was significantly suppressed by curcumin treatment [Fig. 8(A)]. The expression of MMP-9 was found to be approximately 5.3-fold and 4.78-fold of normal mice in the liver of DL and DL+DMSO mice respectively, which was brought down to approximately 71%, 74% and 73% of DL+DMSO mice after curcumin treatment with the dose of 50, 100 and 150 mg/kg bw respectively. Similarly, expression of MMP-2 was elevated upto approximately 3.1-fold and 2.4-fold of normal mice in liver of DL and DL+DMSO mice respectively which was reduced upto approximately 86%, 53% and 53% of DL+DMSO mice with the dose of 50, 100 and 150 mg curcumin/kg bw respectively. The angiogenic potential of MMPs depends upon their protease activity. The analysis of gelatin zymography pattern reveled that MMP-9 was enzymatically active in its pro form (92 kD) as well as in active form (82 kD), whereas only pro form of MMP-2 (72 kD) was observed as enzymatically active [Fig. 8(C)]. The protease activity of pro MMP-9 was approximately 4.2-fold and 4.0-fold while active MMP-9 was approximately 31-fold and 26-fold in the liver of DL and DL+DMSO mice respectively as compared to normal. Curcumin treatment lowered the protease activity towards normal level. Protease activity of pro MMP-9 was found to be approximately 78%, 51% and 67% of DL+DMSO mice, while protease activity of active MMP-9 was found to be approximately 25%, 9% and 25% of DL+DMSO mice with the dose of 50, 100 and 150 mg/kg bw respectively. The protease activity of pro MMP-2 was drastically induced upto approximately 116-fold and 74-fold in liver of DL and DL+DMSO mice respectively. After curcumin treatment the protease activity of pro MMP-2 was approximately 6%, 2%, and 5% of DL+DMSO mice with the dose of 50, 100 and 150 mg/kg bw respectively.

### Hemoglobin content and plug weight of matrigel

**Figure 9:**
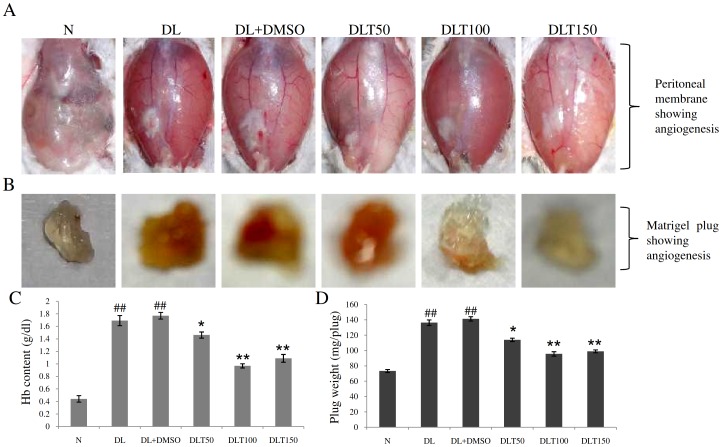
*In vivo* angiogenesis assay. Effect of curcumin on in vivo angiogenesis studied by using Matrigel plug (A) Photographs showing angiogenesis pattern on peritoneal membrane of mouse of different groups, (B) Photographs showing appearance of colour of Matrigels due to vascularisation, removed from different groups, (C) Histogram showing haemoglobin content in Matrigel due to vascularisation (D) Histogram showing weight of Matrigel plug weighed just after removing from different groups. Data represent mean ± S.E.M. #p<0.05 and ##<0.01 compared to N group, *p<0.05 and **p<0.01 compared to DL+DMSO group respectively. N, DL, DL+DMSO, DLT50, DLT100 and DLT150 represents normal, Dalton's lymphoma bearing, Dalton's lymphoma bearing mice treated with DMSO and Dalton's lymphoma bearing mice treated with 50, 100 and 150 mg curcumin/kg body weight dissolved in DMSO respectively.

The progression of cancer is characterized by development of angiogenesis. During angiogenesis blood vessels are entrapped into the matrigel plug placed subcutaneously, owing to increase in level of VEGF in blood [Fig. 9(A and B)]. Thus angiogenesis can be monitored in terms of vascularisation of the plug as shown by measuring the change in plug weight of matrigel plug and hemoglobin content. Hemoglobin content was found to be significantly increased approximately upto 3.9-fold and 3.8-fold in the matrigel plug removed from DL and DL+DMSO mice respectively as compared to normal mice. Hemoglobin content was decreased after curcumin treatment with the doses of 50, 100 and 150 mg/kg bw, which was approximately 82%, 54% and 61% of DL+DMSO mice respectively, suggesting significant decrease in vascularisation of the plug [Fig. 9(C)]. Similarly, plug weight of matrigel removed from DL and DL+DMSO mice was significantly increased upto approximately 1.9-fold and 1.92-fold of normal. Curcumin treatment significantly reduced the plug weight of matrigel indicating reduced vascularisation of the plug. Plug weight of matrigel was observed to be approximately 80%, 67% and 70% of DL+DMSO mice with the dose of 50, 100 and 150 mg/kg bw respectively [Fig. 9(D)].

## Discussion

Carcinogenesis is a multistage process in which several normal cellular pathways go awry. Liver is the second most commonly involved organ by cancer metastasis, after lymph nodes. True prevalence of metastatic liver disease is unknown but, depending on the site of primary tumor, 30–70% of patients dying of cancer have hepatic metastases [27]. Metastatic cells arriving in the liver via circulation encounter hepatic microenvironment. The interactions of tumor cells with hepatic sinusoidal and extra sinusoidal cells determine their fate. However, once the tumor cells survive the initial onslaught, tumors may grow within the liver by reflecting different host responses, vascularisation and proteolytic activity [28]. These three steps are regulated by endogenous proinflammatory factors and reactive oxygen intermediates like superoxide radicals and hydrogen peroxide, released by cancer cells in the hepatic sinusoid microenvironment [27]. We have reported up regulated expression of proinflammatory cytokines like IL-1α, IL-1β, TNF-α and IL-6 in liver DL mice [29], [30]. Here, we observed elevated level of ROS as well as expression and activity of ROS producing enzyme NOX1 and NOX2 in liver of DL mice as compared to normal mice, suggesting oxidative microenvironment in liver of lymphoma bearing mice. Further, PKC is known to generate ROS and PKC-dependent activation of NOX was suggested to be an important mechanism for increased oxidative stress [31]. Upregulation in expression of PKCα as observed in liver of DL mice might induce ROS production via NOX. In our lab, PKCα is reported to be upregulated in liver of DL mice [32], [33]. In addition reduced expression and activities of Mn-SOD and Cu/Zn-SOD further supports the state of oxidative microenvironment in liver of DL mice, as SOD converts highly toxic reactive oxygen intermediate superoxide radical to H_2_O_2_. Reduced expression and activity of Mn-SOD further suggests mitochondrial oxidative stress in liver of DL mice, which drives tumor progression and metastasis [34]. Apart from this, higher level of H_2_O_2_ in liver of DL mice was also observed, as major part of H_2_O_2_ production in cancer condition is contributed by peroxisomes [35]. Production of H_2_O_2_ is reported to increases in both liver cells and tumor cells during the initial stages of tumor metastasis that regulate NF-κB [36]. Reduced expression and activity of H_2_O_2_ detoxifying antioxidant enzymes catalase and glutathione peroxidase are reported previously by us in liver of DL mice, which favors accumulation of H_2_O_2_ [19]. Taken together, these changes in liver of DL mice confirm depleted antioxidant defence system. Decrease in level of ROS as well as H_2_O_2_ via down regulation of expression and activity of NOX and upregulation of expression and activity of SOD by curcumin treatment to DL mice provides substantial evidence that Mn-SOD behaves as a potent tumor suppressor protein [37], [38]. Overexpression of Mn-SOD reduces tumorigenicity and metastatic capability of many tumor types [39]. NADPH oxidase-dependent ROS generation was shown to be involved in cytoskeletal remodelling, extravasation, angiogenesis and regulation of genes associated with tumor metastasis [40]. Down regulation of expression of PKCα by curcumin also supports reduction in ROS production. Thus, reduced ROS production brought about by curcumin might be resulted in inhibition of metastasis, as targeted delivery of antioxidant enzymes has been demonstrated to inhibit tumor metastasis [41].

Reduced level of antioxidant defence in oxidative tumor microenvironment is associated with deregulation in expression of oncogenes like cMyc. Besides, hypoxia prevalence in hepatic metastases of greater than 300 µm in diameter and new intra tumoral capillaries are established in micrometastases [27]. Upregulated expression of cMyc and HIF-1α in liver of DL mice further confirmed carcinogenic activity in hypoxic tumor microenvironment of liver of DL mice. Deregulation of c-Myc occurs in nearly 30% of human cancers. Under physiologic conditions, HIF inhibits c-Myc activity; however when deregulated, oncogenic c-Myc collaborates with HIF in inducing the expression of PDK1, HK2 and VEGF [42]. The activation of HIF-1 is critical for tumor cell survival and proliferation in hypoxic tumor microenvironment [8]. HIF-1 in collaboration with cMyc takes major role in regulation of cancer cell metabolism. Cancer cells consume a larger amount of glucose, maintain a much higher rate of glycolysis and convert majority of glucose into lactic acid even in the presence of oxygen compared to that of normal cells. Activation of glycolytic genes by HIF-1 is considered critical for metabolic adaptation to hypoxia through increased conversion of glucose to pyruvate and subsequently to lactate. In addition, HIF-1α suppresses metabolism through TCA cycle by directly trans-activating the PDK1 [43]. Further, increase in lactate formation is accompanied by increased expression and activity of LDH-A, which is encoded by a target gene of cMyc and HIF-1α [7]. Elevated expression and activity of LDH-A in DL mice compared to normal mice suggests higher production of lactic acid in the liver of DL mice, as reported previously in our lab [44]. LDH-A plays a key role in tumor maintenance. Attenuation of LDH-A expression is reported to uncover a link between glycolysis, mitochondrial physiology, and tumor maintenance [45]. Anticarcinogenic capacity of curcumin is shown in our study by down regulation of expression of HIF-1α and cMyc towards normal level. Antitumorigenic effect of antioxidants is reported to be dependent on HIF-1α *in vivo* [46]. Our result of decreased expression and activity of LDH-A, by curcumin treatment suggests inhibition of cellular transformation and *in vivo* tumorigenesis as reported earlier [47].

HIF-1 enhances oxygen delivery via angiogenesis under hypoxic tumor microenvironment. Expression of VEGF, an important angiogenic factor is regulated by HIF-1 in response to hypoxia. Expression of VEGF is reported to be upregulated in mouse model of experimental liver metastasis [48]. Further, upregulated mRNA expression as well as protein level of VEGF-A in liver of DL mice suggest that HIF-1α and dysregulated cMyc cooperatively induce the expression of VEGF-A in hypoxic microenvironment for neovascularisation [8]. In addition, elevated expression of PKCα might promote angiogenic activity of VEGF, as endothelial cells actively produce VEGF in response to PKCα and VEGF in turn promotes its sustained release via an autocrine positive feedback loop [2]. Down regulated expression of VEGF-A both at mRNA and protein level by curcumin treatment confirms regulation of VEGF-A via HIF-1α [49]. In addition, modulation of VEGF might be supported by PKCα. MMPs play an active role in reactivation of VEGF as well as proteolytically degrade various components of the extracellular matrix (ECM) necessary for degradation of vascular basement membrane and remodelling of ECM for migration and invasion of endothelial cells into the surrounding tissue. Specific MMPs have been shown to enhance angiogenesis via detachment of pericytes from vessels undergoing angiogenesis, release of ECM-bound angiogenic growth factors, exposure of cryptic pro angiogenic integrin binding sites in the ECM, generation of pro migratory ECM component fragments and splitting of endothelial cell-cell adhesions [50]. MMPs especially MMP-9 and -2 are reported to be involved in tumor angiogenesis mainly through their degradative capacity [51]. Abruptly higher expression and activity of MMP-9 and -2 suggests metastatic and angiogenic environment in liver of DL mice. MMP2 was reported to be highly expressed by endothelial cells in different tumors including human glioblastomas and its activation is contributed by H_2_O_2_, which is found highly elevated in liver of DL mice. MMP-9 participates in the degradation of basement membrane during the initial phase of angiogenesis [52]. Further, progression of angiogenesis was also confirmed by development of blood vessels on peritoneal membrane, as well as by *in vivo* angiogenesis shown by significant increase in vascularisation of matrigel plug. Decreased expression and activity of MMP-2 and -9 towards normal as well as decreased vascularisation of matrigel plug by curcumin treatment reflects modulation of hypoxic microenvironment and inhibition of angiogenesis.

Considering different parameters studied here, optimal dose of curcumin was found 100 mg/kg body weight. Lower dose of 50 mg/kg body weight might not be sufficient to produce optimal effect and higher dose of 150 mg/kg body weight may cause negative feedback regulation.

In summary, the findings presented herein highlight the elevated expression of HIF-1α and cMyc in oxidative microenvironment of liver of DL mice with lower antioxidant defence status, which expedites angiogenesis via inducing glycolytic metabolism and upregulation of VEGF mediated by PKCα and through activation of MMP2 and 9. Long term effect of curcumin reflects its anticarcinogenic action by inhibiting angiogenic activities via modulation of glycolytic pathway, activation of endogenous antioxidant defense system and inhibition of ROS formation in liver of lymphoma bearing mice, which suggest antioxidant and anti-inflammatory property of its metabolites. Thus findings of this study suggests that anticarcinogenic action of curcumin, even after withdrawal of treatment might be due to synergistic action of its metabolites as metabolism of curcumin is very fast. However, this needs further verification using different isolated metabolites.
